# Coexpression of adrenomedullin and its receptor component proteins in the reproductive system of the rat during gestation

**DOI:** 10.1186/1477-7827-8-130

**Published:** 2010-10-29

**Authors:** Lei Li, Fai Tang, Wai-Sum O

**Affiliations:** 1 Department of Physiology, Li Ka Shing Faculty of Medicine, The University of Hong Kong, Pokfulam, Hong Kong SAR, China; 2 Department of Anatomy, Li Ka Shing Faculty of Medicine, The University of Hong Kong, Pokfulam, Hong Kong SAR, China; 3 Centre of Heart, Brain, Hormone and Healthy Aging, Li Ka Shing Faculty of Medicine, The University of Hong Kong, Pokfulam, Hong Kong SAR, China; 4 Centre of Reproduction, Development and Growth, Li Ka Shing Faculty of Medicine, The University of Hong Kong, Pokfulam, Hong Kong SAR, China

## Abstract

**Background:**

Adrenomedullin (ADM), a novel vasorelaxant peptide, was found in human/rat ovaries and uteri. Plasma ADM level increases in pregnant women and pregnant rats.

**Methods:**

The gene expression levels of *Adm *and its receptor components - *Crlr*, *Ramp1*, *Ramp2 *and *Ramp3*, the ADM peptide concentration and localization in the rat female reproductive system during gestation were studied by real-time RT-PCR, EIA and immunohistochemical techniques.

**Results:**

The mRNAs of *Adm *and its receptor component and ADM were differentially distributed between implantation sites and inter-implantation sites of the pregnant uterus. The day on which vaginal sperm were found was taken to be pregnancy day 1. The *Adm *mRNA levels in the implantation sites of the uteri in mid- (day 12) and late pregnancy (day 17) were more than 10-fold higher than those in nonpregnancy, pre-implantation (day 3) or early (day 7) pregnancy. ADM was localized in the endometrial stroma with increased immunoreactivity from nonpregnancy to pregnancy. The ADM level and the mRNA levels of *Adm*, *Crlr*, *Ramp2 *and *Ramp3 *in the corpus luteum all increased in late pregnancy compared with early pregnancy. The gene expression of *Adm *and it receptor components and intense immunostaining of ADM were also found in the oviduct during pregnancy.

**Conclusions:**

The gene expressions levels of *Adm *and its receptor components - *Crlr, Ramp1, Ramp2 *and *Ramp3*, and ADM peptide concentration exhibited a spatio-temporal pattern in the rat female reproductive system during gestation and this suggests that ADM may play important roles in gestation.

## Background

Adrenomedullin (ADM) was a novel potent vasodilator first discovered in human pheochromatocytoma tissue in 1993 [1]. The *Adm *gene and its protein product are highly conserved across species, including human, rat, pig and cow [2-5]. It is a member of the calcitonin family with a high sequence homology to calcitonin gene-related peptide (CGRP). It consists of 52 amino acids in human and 50 amino acids in the rats [2,3]. ADM can bind to the CGRP receptor in several types of tissues [6,7], but specific ADM receptors that are insensitive to CGRP receptor antagonist have been identified [8]. McLatchie et al. [9] demonstrated that the combination of calcitonin receptor-like receptor (CRLR) and receptor activity-modifying protein (RAMP) isoforms determines the ligand selectivity for CGRP and ADM. Coexpression of CRLR with RAMP1, RAMP2 and RAMP3 produces the CGRP, the ADM1 and ADM2 receptors respectively.

ADM is synthesized and secreted by various organs and tissues, including the heart, kidney, lung, adrenal gland and reproductive organs, such as the ovary [10-14], the uterus [10,15], the oviduct [14], the testis [16-19], the prostate [20,21], and the epididymis [22]. Since its discovery as a potent vasodilator, there have been extensive studies on the pleiotropic effects of ADM [23-26]. Remarkable increases in plasma ADM concentrations were reported in both in human [27] and the rat [28] during pregnancy. ADM found in granulosa cells enhances progesterone production in human [13] and suppresses FSH-stimulated granulosa cell differentiation in the rat [11] by an autocrine action. The increases in ADM expression and binding sites in the rat uterus in late gestation [15] may be related to the inhibitory role of ADM in uterine contractility [15,29]. The infusion of an ADM antagonist (hADM22-52) during early or late gestation in the rats caused fetoplacental growth restriction [30,31]. The *Adm-/- *mice are non-viable [32-34]; and the haploinsufficient (*Adm*+/-) female mice were reported to have reduced pregnancy success [35], in support of a highly essential role for ADM in gestation especially in fetal growth and development. The expression of ADM and its receptors in the ovary and the oviduct of the cycling rats has been studied by our group and ADM was found to inhibit FSH-induced estradiol secretion from follicles and eCG-stimulated progesterone release in corpora lutea [14]. We hypothesize that the distribution and levels of ADM and its receptors may change at different stages of pregnancy according to the roles of ADM. The expression of ADM and its receptors in the uterus, the oviduct and the corpus luteum in the rats of early, mid- or late pregnancy was therefore studied for possible physiological roles of ADM during gestation.

## Methods

### Animals

Mature Sprague-Dawley rats (12-13 week old) were obtained from the Laboratory Animal Unit, LKS Faculty of Medicine, the University of Hong Kong. Female rats showing three consecutive regular oestrous cycles were included in this study. The rats were housed at a constant temperature, humidity, under a 12-hour light-dark cycle (dark period 07:00 h to 19:00h) and rat chow and water were available ad libitum. Proestrus females were caged with proven fertile males overnight. The presence of vaginal sperm the following morning was taken evidence of pregnancy and this was day 1 of pregnancy. Rats of pre-implantation, early, mid- and late pregnancy were killed on days 3, 7, 12 and 17 of gestation. Pseudopregnant females obtained by mating with vasectomized male rats were also killed on day 7 of pseudopregnancy. Diestrus females were killed as nonpregnant controls. The uteri were dissected and implantation sites and inter-implantation sites were separated on days 7, 12 and 17 pregnancy. The foetuses and placentae of day 12 and 17 pregnant rats were collected before the dissection of the uteri. The ovaries were collected and the corpora lutea were isolated under a dissecting microscope using two 26G syringe needles. The oviducts were also collected and all the tissues were snap-frozen in liquid nitrogen and stored at -80°C for further analysis. Freshly collected intact uteri, ovaries and oviducts were fixed in neutral buffered formalin for immunohistochemical study. All procedures were approved by the Committee on the Use of Live Animals for Teaching and Research, the University of Hong Kong.

### Genes expression of *Adm*, *Crlr*, *Ramp1*, *Ramp2 *and *Ramp3*

Total RNA of the uterus, corpus luteum, oviduct, foetus and placenta were extracted using TRIZOL reagent and subjected to real-time RT-PCR analysis. RNA samples (1 μg) were reverse transcribed into cDNA with the iScript reverse transcriptase according to the manufacturer's instructions (Bio-Rad Laboratories, Hercules, CA). The real-time RT-PCR technique has been previously described [15]. Standard curves for each primer pair were prepared using serial dilution of the cDNA to determine PCR efficiency. The PCR efficiencies for *Adm*, *Crlr*, *Ramp1*, *Ramp2*, *Ramp3 *and *Actb *were all above 0.95 and similar. The relative gene expression levels normalized to *Actb *were analyzed using the ΔΔCT method, where CT was the cycle threshold. The reaction mixtures contained 25 μl iQ SYBR Green Supermix (Bio-Rad Laboratories, Hercules, CA), 500 nM of each primer, 1 μl template cDNA, and DNase-free water to a final volume of 50 μl. Cycle conditions were 95°C for 10 min, followed by 40 cycles of 95°C for 45 sec, 59°C for 30 sec, and 72°C for 45 sec. The reaction was completed with a dissociation step for melting point analysis from 50°C to 95°C (in increments of 0.5°C for 10 sec each. The primers were designed on the basis of the published sequences of *Adm *(caggacaagcagagcacgtc, forward; tctggcggtagcgtttgac, reverse); *Crlr *(ccaaacagacttgggagtcactagg, forward; gctgtcttctctttctcatgcgtgc, reverse); *Ramp1 *(cactcactgcaccaaactcgtg, forward; cagtcatgagcagtgtgaccgtaa, reverse); *Ramp2 *(aggtattacagcaacctgcggt, forward; acatcctctgggggatcggaga, reverse); *Ramp3 *(acctgtcggagttcatcgtg, forward; acttcatccggggggtcttc, reverse) and *Actb *(ggaaatcgtgcgtgacatta, forward; aggaaggaaggctggaagag, reverse). Melt curve analysis for each primer set revealed only one peak for each product. The size of the PCR products was confirmed by comparing the size of the product with a commercial ladder after agarose gel electrophoresis.

### Immunocytochemistry (IHC)

Immunohistochemistry was performed on 5 μm sections of paraffin-embedded tissues with a peroxidase-labeling kit (Vector Laboratories, Burlingame, CA). The antiserum against rat ADM (Phoenix Pharmaceuticals, Burlingame, CA) was used at a final dilution of 1:500. Staining was visualized using a DAB substrate kit for peroxidase (Vector Laboratories, Burlingame, CA). The antibody was omitted from control sections to check for nonspecific staining.

### Extraction of ADM from the uterus, ovary, oviduct, foetus and placenta

Tissues including the uterus, corpus luteum, oviduct, foetus and placenta were homogenized in 2N ice-cold acetic acid and then boiled for 10 min. A 50 μl aliquot was taken for Bio-Rad protein assay (Bio-Rad Laboratories, Hercules, CA). The remaining homogenates were centrifuged at 18, 600 × g for 20 min at 4°C. The supernatants were lyophilized and stored at -20°C.

### ADM Enzyme Immuno Assay (EIA)

The lyophilized tissue samples were reconstituted in 1X ADM EIA assay buffer. ADM peptide concentrations were measured in duplicates using an ADM (1-50) (rat) EIA kit (Phoenix Pharmaceuticals, Burlingame, CA). The minimum detectable concentration was 0.15 ng/ml and the range was 0-100 ng/ml. The intra-assay and inter-assay errors were less than 5% and 14% respectively.

### Statistical analysis

The data are expressed as mean ± SEM. When there was only one variable, One-way ANOVA with the Fisher's Least Significance Difference post-hoc test was employed. When there were two variables, 2-way ANOVA was used. The significance level was set at P < 0.05.

## Results

### Expression of Adm and its receptor components in the uterus, foetus, placenta, corpus luteum and oviduct during pregnancy

**Figure 1:**
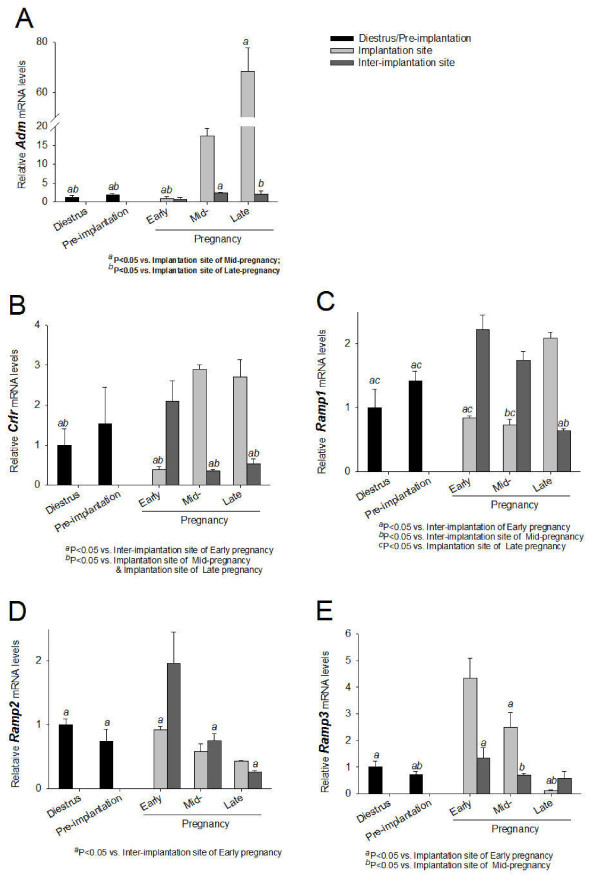
**Expression of *Adm *and its receptor components in the uterus from early, mid- and late pregnant rat**. Expression of *Adm *(A), *Crlr *(B), *Ramp1 *(C), *Ramp2 *(D) and *Ramp3 *(E) in the uterus from nonpregnant rats and rats of different pregnancy stages. Data were presented as mean ± SEM. n = 3 for nonpregnancy, and pre-implantation, n = 6 for implantation sites and inter-implantation sites of early, mid- and late pregnancy.

The results for the mRNA levels of *Adm *and its receptor components, *Crlr, Ramp1, Ramp2 *and *Ramp3 *were shown in Figure 1. In the uterus, *Adm *was differentially expressed between implantation and inter-implantation sites. *Adm *mRNA levels at the implantation sites of mid- (12-day) and late (17-day) pregnant rats were much higher than those of the inter-implantation sites (Figure 1A), which had values similar to those in the uteri of nonpregnant, pre-implantation (3-day) and early (7-day) pregnant rats (Figure 1A). The *Adm *mRNA levels of the uteri from the pseudopregnant rats were 80% of those from the nonpregnant rats (0.80 ± 0.10, n = 11 vs 1.00 ± 0.08, n = 14) and the values were not significantly different. There were also increases in *Crlr *(Figure 1B) and *Ramp1 *(Figure 1C) mRNA levels in implantation sites at late pregnancy compared with early pregnancy. For the *Ramp2 *(Figure 1D) and *Ramp3 *(Figure 1E) mRNA levels, there was a trend for a decrease from early to late pregnancy in both the implantation and inter-implantation sites. The mRNA levels of ADM receptor component proteins were also differentially expressed (Figure 1B-E). During early pregnancy, the expression levels of *Crlr, Ramp1 *and *Ramp2 *were all higher at inter-implantation sites than implantation sites (Figure 1B-D) and the reverse was true for *Ramp3 *(Figure 1E). During late gestation, the relative expression levels of *Crlr, Ramp1, Ramp2 *and *Ramp3 *between implantation sites and inter-implantation sites were opposite to those in the early gestation, with higher levels of *Crlr, Ramp1 *and *Ramp2 *at implantation sites (Figure 1B-D) and higher *Ramp3 *level at inter-implantation sites (Figure 1E). During mid-gestation, the expression levels of both *Crlr *and *Ramp3 *at the implantation sites were higher than inter-implantation sites and that of *Ramp1 *were lower at the implantation sites (Figure 1B-E).

**Figure 2:**
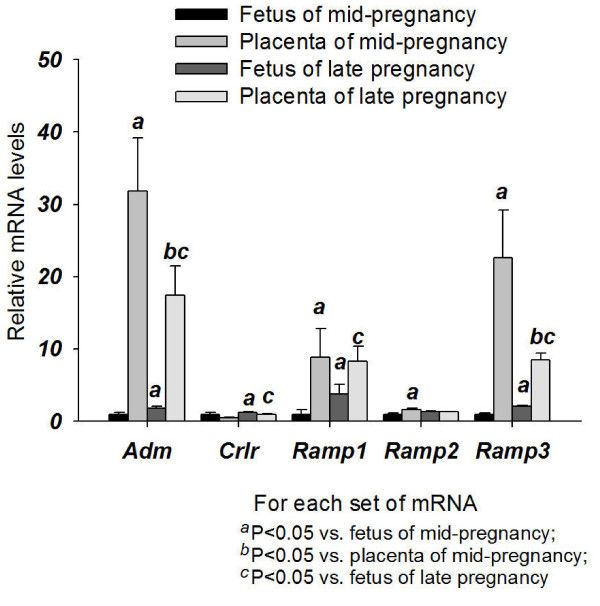
**Expression of *Adm *and its receptor components in the foetuses and placentae**. Expression of *Adm, Crlr, Ramp1, Ramp2 *and *Ramp3 *in the foetuses and placenta from mid- and late pregnancy. Data were presented as mean ± SEM. n = 6-8 for foetus, n = 6 for placenta.

The gene expression levels of *Adm*, *Ramp1 *and *Ramp3 *of the placenta were higher than those of the foetus in both mid- and late gestation (Figure 2). The mRNA levels of *Adm *and *Ramp3 *in the placenta were higher in mid-gestation than in late gestation. The foetus had a higher *Adm*, *Crlr*, *Ramp1 *and *Ramp3 *mRNA levels in late gestation than mid-gestation (Figure 2).

**Figure 3:**
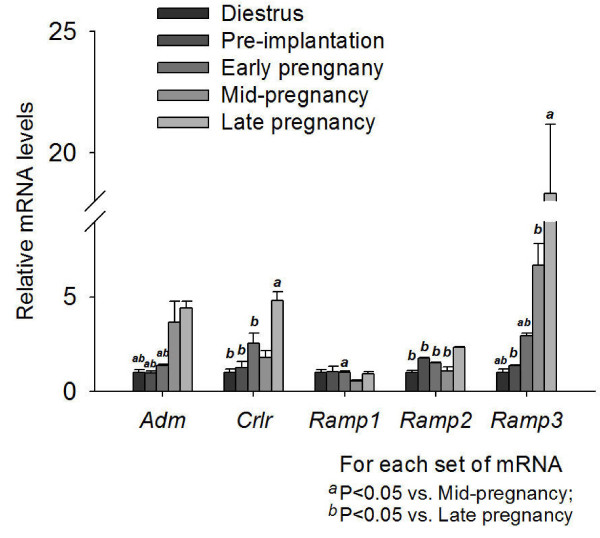
**Expression of *Adm *and its receptor components in the corpus luteum from early, mid- and late pregnant rat**. Expression of *Adm*, *Crlr*, *Ramp1*, *Ramp2 *and *Ramp3 *in the corpus luteum from nonpregnant rats and rats of different pregnancy stages. Data were presented as mean ± SEM. n = 3.

In the corpus luteum (Figure 3), the *Adm *mRNA levels were dramatically increased in mid- and late gestation. The mRNA levels of receptor components *Crlr, Ramp2 *and *Ramp3 *in late gestation were also elevated. The mRNA levels of *Ramp3 *showed a continual increase from nonpregnancy to late gestation.

**Figure 4:**
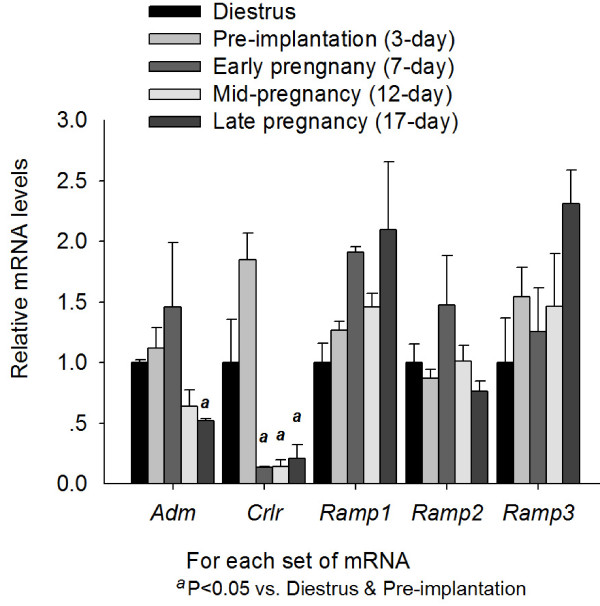
**Expression of *Adm *and its receptor components in the oviduct from early, mid- and late pregnant rat**. Expression of *Adm, Crlr, Ramp1, Ramp2 *and *Ramp3 *in the oviduct from nonpregnant rats and rats of different pregnancy stages. Data were presented as mean ± SEM. n = 6-7.

In the oviduct (Figure 4), the expression levels of *Adm *decreased from pre-implantation to late pregnancy. The expression levels of *Crlr *were much lower from early to late gestation than pre-implantation. There was no significant difference in the expression levels of *Ramp1, Ramp2 *or *Ramp3*.

### ADM peptide levels in the uterus, foetus, placenta, corpus luteum and oviduct during pregnancy

**Figure 5:**
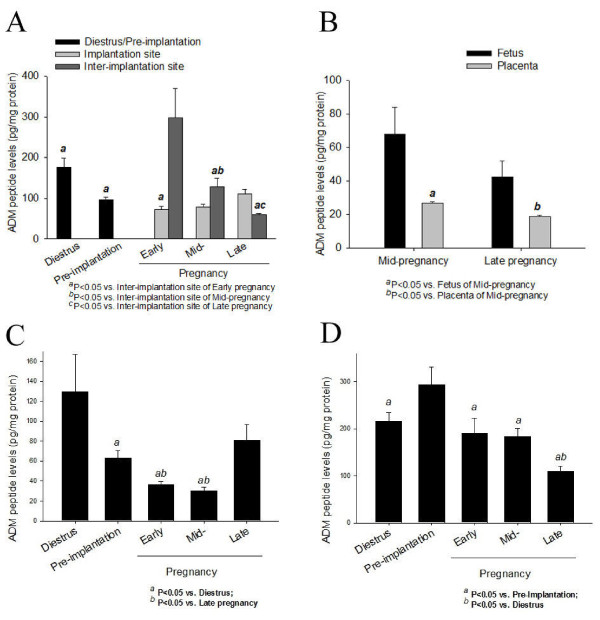
**ADM peptide levels in the uterus, foetus and placenta, corpus luteum, and oviduct**. ADM peptide levels in the uterus (A) from nonpregnant rats and rats of different pregnancy stages, in the foetus and placenta from mid- to late pregnant rats (B), and in the corpus luteum (C), oviduct (D) from nonpregnant rats and rats of different pregnancy stages. Data were presented as mean ± SEM. n = 3-9.

In the uterus, ADM was differentially distributed between the implantation sites and inter-implantation sites in both early and late gestation (Figure 5A). While there was no differences between the uterus samples from nonpregnant (234 ± 12 pg/mg protein, n = 15) and pseudopregnant (230 ± 19 pg/mg protein, n = 12) rats, the ADM level was higher in inter-implantation sites than implantation sites in early and mid-pregnancy and the opposite was true in late pregnancy (Figure 5A). Overall, the inter-implantation sites in early pregnancy had the highest level of ADM peptide. The ADM levels in the inter-implantation sites in early pregnancy declined at mid- and late pregnancy. The foetuses from mid-pregnant rats showed a higher ADM level than the placentae from mid-pregnant rats (Figure 5B). The placental ADM levels decreased from mid-gestation to late gestation (Figure 5B). The ADM levels in the corpora lutea of nonpregnant and late pregnancy rats were higher than those from early and mid-pregnant rats (Figure 5C). The ADM levels of the oviduct were higher at pre-implantation and decreased in late pregnancy (Figure 5D).

### Immunohistochemical study of ADM in the female reproductive system

**Figure 6:**
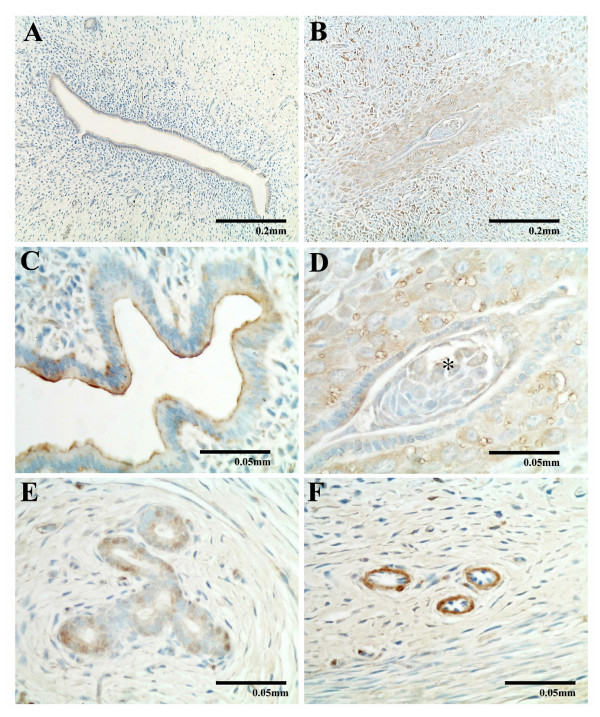
**Immunocytochemical study of ADM in the uterus from nonpregnant and early pregnant rats**. Immunocytochemical study of ADM in the rat uterus from nonpregnant (A) and early pregnant rats (B-F) showing positive ADM immunoreactivity localized to the decidua (B), the luminal epithelium (C), the embryo (asterisk, D), glandular epithelial cells (E) and endothelial linings of the blood vessels (F) of the uterus from early pregnant rats.

The nonpregnant uterus showed ADM positive staining at endometrium (Figure 6A) and weak staining at myometrium and serosa (data not shown). The uteri of early pregnant (7-day) rats with implanted embryos showed heavier ADM staining than those of nonpregnant rats, especially in the decidua (Figure 6B). The embryo was also positive for ADM (Figure 6D). In the endometrium, ADM staining was also found in the luminal epithelium (Figure 6C), the glandular epithelia cells (Figure 6E) and the endothelial lining of the blood vessels (Figure 6F).

**Figure 7:**
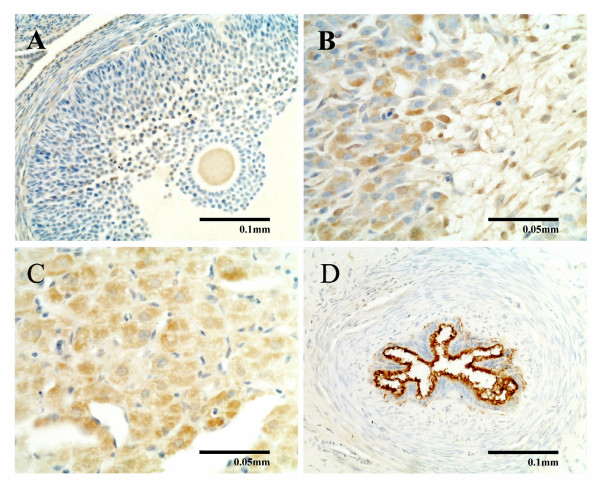
**Immunocytochemical study of ADM in rat ovary and oviduct**. Immunocytochemical study of ADM in the ovary (A, B, C) and oviduct (D) showing positive ADM immunostaining in the follicles (A) and corpora lutea (B). Intense ADM immunostaining was found at the centre of some corpora lutea from late pregnant rats (C) and oviduct (D).

In the ovary from the nonpregnant rats, ADM positive staining was localized to interstitial cells, ovum, cumulus oophorus and granulose cells of the follicles (Figure 7A) and luteal cells of the corpus luteum (Figure 7B). In the pregnant rats, the majority of the ovarian volume was taken up by the growing corpora lutea, which were also the main location of ADM staining. There was no obvious difference in the density of ADM staining in the corpus luteum from nonpregnant rats to pregnant rats (data not shown), though there was heavy ADM staining at the centre of some corpora lutea from late pregnant rats (Figure 7C).

In the oviduct, intense ADM staining was localized in the mucosal layer of the oviduct, especially in the ciliated epithelium from nonpregnant and pregnant rats (Figure 7D). The muscularis and serosa layers of the oviduct showed little ADM staining.

There was no staining in the negative controls (see Additional file 1, Figure S1).

## Discussion

To the best of our knowledge this is the first time course study of ADM and its receptor component proteins in pregnancy in the female reproductive tract and corpus luteum. In our previous study in the rat, the oviduct expressed the highest level of ADM in the female reproductive tract during the oestrous cycle [14]; but ADM and its mRNA have not been characterized in the pregnant rats. ADM was most intense in the ciliated epithelium. CGRP is known to inhibit contractility in the human oviducts [36] and CGRP-binding sites are found on the vascular smooth muscle cells [37]. Endothelin 2 has been shown to stimulate oviductal contraction in the rat [38] and ADM may inhibit this effect (Liao, Ho, Tang and O, unpulished data). Recently, we have also demonstrated that the presence of sperm in the oviduct stimulates ADM production, which in turn stimulates ciliary beating in the rat [39] and human [40] oviducts. During pregnancy, the increase in ADM peptide in the pre-implantation period may be related to the presence of sperm in the oviduct as well as its inhibitory action on oviductal contraction, which facilitate the ova/embryo transportation into the uterus. The decreases in ADM peptide and *Adm *mRNA levels in late pregnancy and in *Crlr *mRNA in mid- and late pregnancy may indicate that this inhibition is less important after placenta formation.

The most dramatic changes in ADM peptide and mRNA expression occurred in the uterus during pregnancy. Both the *Adm *mRNA and ADM were differentially distributed between implantation and inter-implantation sites. The lack of a difference in *Adm *mRNA levels and ADM peptide levels between the nonpregnant uterus and the pseudo-pregnant uterus suggests that such changes in the implantation sites must be due to the contact between the embryo and the uterus instead of alterations in hormone levels in the systemic circulation. In early gestation, ADM levels and mRNA levels of *Crlr, Ramp1*, and *Ramp2 *in the inter-implantation sites were higher than in implantation sites, in line with a role for ADM to increase blood supply [41] and to inhibit uterine contractility [42]. *Adm *mRNA levels in the implantation sites of the uteri from mid- and late pregnant rats were much higher than the inter-implantation sites. The differences were much less marked in ADM peptide levels, suggesting a possible increase in release. An increase in *Adm *mRNA level in the uteri of pregnant rats has been previously reported [14]. Plasma ADM levels are elevated in pregnant rats [28,43] and pregnant women [27], presumably due to secretion by the uterus [14,41] for the adaptation of the vascular system to pregnancy [44]. The very high level of *Adm *mRNA and high levels of *Crlr *and *Ramp1 *mRNA at late pregnancy suggests an increasingly important role of ADM towards term.

The gene expression levels of *Crlr *and *Ramp1 *were higher at the implantation sites of late pregnancy than those in the uteri of nonpregnant rats, in line with a previous report of an increase in ADM binding sites in late pregnancy [14]. This change may increase the number of CGRP receptors and augment the action of ADM, the expression of which was also increased. It is pertinent to note that ADM has been shown to inhibit uterine contraction via the CGRP receptor [14]. The increase in *Crlr *and *Ramp1 *gene expression from early to late pregnancy is also in line with the increase in CGRP receptor binding from day 7 of gestation [45]. CGRP is found in the nerves in the uterus [46] and inhibits uterine contraction [47] and causes vasorelaxation of the uterine artery [48].

Active angiogenesis is observed during early to mid-pregnancy to meet the need for an increase in blood supply [49]. ADM have been proposed to exhibit its pro-angiogenic actions through CRLR/RAMP2 and CRLR/RAMP3 receptors [50] or CRLR/RAMP2 receptor [51]. The high *Crlr*, *Ramp2 *and *Ramp3 *expression levels in the uterus in early and mid-pregnancy might contribute to the increased action of ADM in angiogenesis. Immunohistochemical study showed that ADM were located mainly in the endometrial stroma, similar to the finding of Cameron et al. [10,52]. In our study, a trend for an increase in ADM immunoreactivity was observed from nonpregnant uterus to the pregnant uterus. The nonpregnant and 3-day pregnant uteri showed limited ADM staining in the epithelial cells lining the uterus while in early pregnancy, stronger staining was localized not only in the epithelium, but also in the blood vessels. The intense ADM immunostaining in the blood vessels suggests both an angiogenic role [42] and a vasodilatory role [43] in the endometrial blood vessels for increasing blood supply. ADM may have multiple roles in the uterus, regulating angiogenesis and blood flow in early pregnancy and maintaining uterine quiescence at mid- and late pregnancy. Studies involving the infusion of ADM receptor antagonist in early [31] and late [30] gestation have also demonstrated the importance of ADM in placentation and embryo development. The infusion of the ADM2 receptor antagonist in late pregnancy produced similar results [53].

Both the levels of ADM peptide and *Adm *mRNA in the placenta were lower in late pregnancy than mid-pregnancy. The mRNA levels of *Ramp3 *also showed a similar decline. To the best of our knowledge, this finding has not been described before in the rat placenta. In human, the concentrations of *ADM *mRNA in the placenta were found to be greater at term than in the first trimester [54]. The significance of these changes remains an enigma. The mRNA levels of *Adm*, *Crlr*, *Ramp1 *and *Ramp3 *in the foetus were higher in late pregnancy than mid-pregnancy. This is related to the importance of ADM in embryogenesis as ADM is involved in embryonic invasion, proliferation, and differentiation, based on the developmental expression patterns of ADM in the rat and mouse embryos [55]. The *Adm *gene expression in the placenta was higher while the peptide levels were lower than those in the foetus in mid- and late pregnancy, suggesting an increase in ADM release from the placenta. Human placenta has indeed been proposed to be the source of foetal circulating ADM as the ADM concentration in the umbilical vein was higher than in the artery [27].

The corpus luteum was studied instead of the entire ovary because it contains most of the ADM, as reported here and by Li et al. [14]. The mRNA levels of *Adm*, *Crlr *and *Ramp2 *and *Ramp3 *in the corpus luteum all increased in late pregnancy compared with nonpregnancy, pre-implantation and early pregnancy and ADM peptide levels also increased at late pregnancy compared with early and mid-pregnancy. These increases may be related to the postulated roles of ADM in anti-luteolysis [12] and the production of progesterone [14]. ADM appears to play a double role in corpus luteum progesterone production in that it is inhibitory in early and late pregnancy but stimulatory in mid-pregnancy (Li et al. unpublished data). The stimulatory effect of ADM on steroidogenesis has been reported in human granulosa-lutein cells [13].

## Conclusions

In summary, there is a spatio-temporal pattern for the gene expression levels of *Adm *and its receptor components - *Crlr, Ramp1, Ramp2 *and *Ramp3*, the ADM peptide concentration and localization in the rat female reproductive system during gestation. In the oviduct, an increase in ADM peptide was found only in the preimplantation stage when the gametes/embryos were present in this part of the reproductive tract. During early pregnancy, the contact of embryo in the implantations sites upregulated ADM peptide and mRNA expression. The increase was further augmented in mid-pregnancy and similar increase was found in the placentae and the expression was comparatively low in the foetuses. A dramatic increase in ADM peptide and mRNA expression was also found in the corpus luteum during mid- and late pregnancy. All these changes suggest important roles for ADM in the maintenance of pregnancy as well as embryogenesis.

## Competing interests

The authors declare that they have no competing interests.

## Authors' contributions

LL worked on mating of rats, collection of tissues, real time RT-PCR, ELISA and drafted the manuscript. WSO and FT coordinated the project. WSO and FT are respectively principal and co-investigators of the research and holders of the grant. Both WSO and FT helped in the revision of the manuscript. All authors read and approved the final manuscript.

## Supplementary Material

Additional file 1 **Figure S1**. Negative controls of ADM Immunocytochemical study. Click here for file
