# Supplementary material for: Coexpression of adrenomedullin and its receptor component proteins in the reproductive system of the rat during gestation
Source: Reprod Biol Endocrinol. 2010 Oct 29;8:130. doi: 10.1186/1477-7827-8-130 (PMC2984462; doi:10.1186/1477-7827-8-130)
Supplement: Additional file 1 — Figure S1. Negative controls of ADM Immunocytochemical study. [file 1477-7827-8-130-S1.PDF]

**Supplemental figure 1**

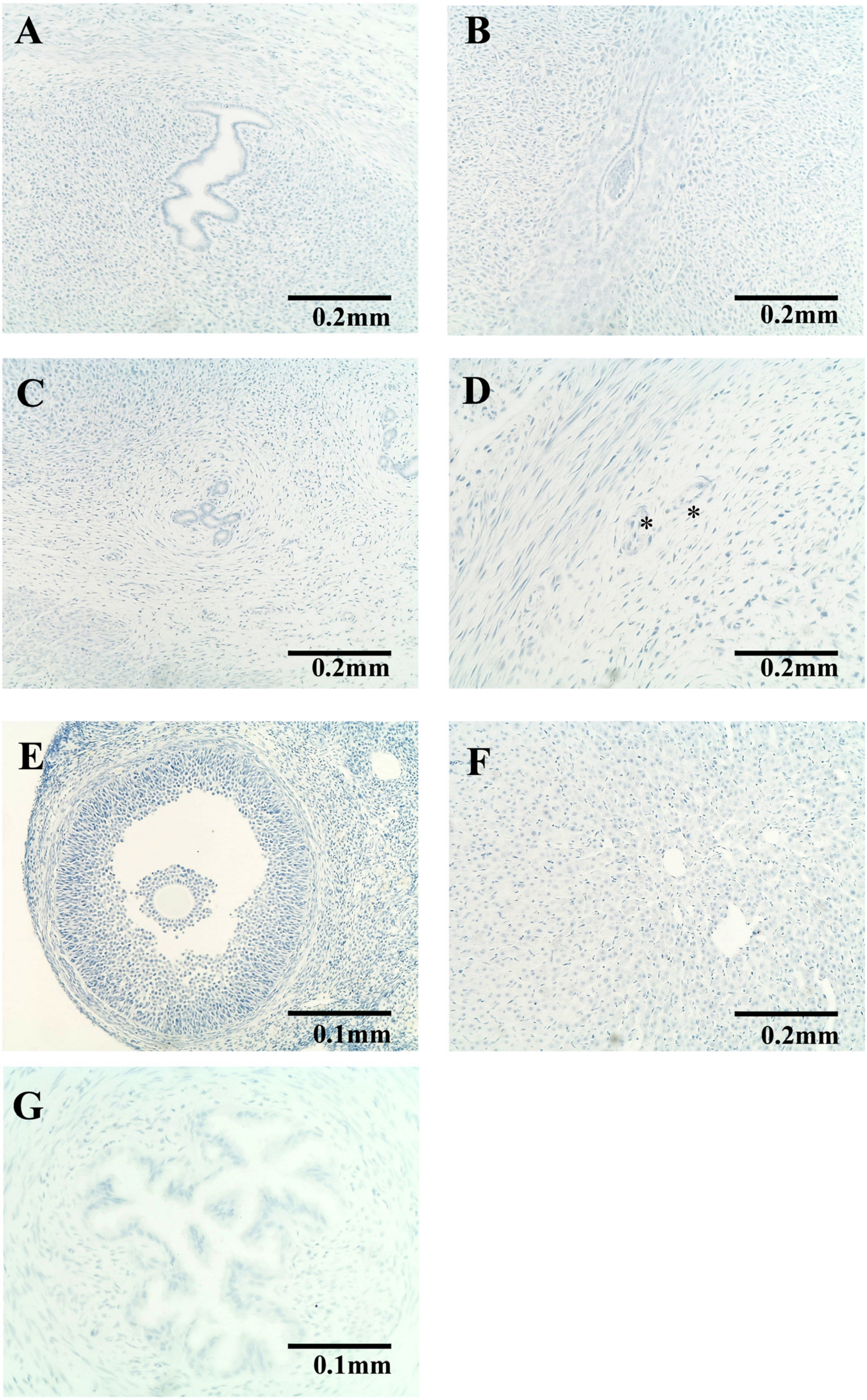


Negative controls of ADM Immunocytochemical study for the uterine lumen (A), the implanted embryo (B), uterine gland (C) and blood vessels (D, asterisks) in the 7-day pregnant uterus; follicle (E), corpus luteum (F) and oviduct (G).
